# Supplementary material for: Transcriptional Interference Gates Monogenic Odorant Receptor Expression in Ants
Source: bioRxiv. 2025 Aug 21:2025.08.21.671318. Preprint. [Version 1] doi: 10.1101/2025.08.21.671318 (PMC12393541; doi:10.1101/2025.08.21.671318)
Supplement: 1 [file NIHPP2025.08.21.671318V1-supplement-1.pdf]

bioRxiv preprint doi: https://doi.org/10.1101/2025.08.21.671318; this version posted August 21, 2025. The copyright holder for this preprint (which was not certified by peer review) is the author/funder, who has granted bioRxiv a license to display the preprint in perpetuity. It is made available under aCC-BY 4.0 International license.

SUPPLEMENTAL FIGURES

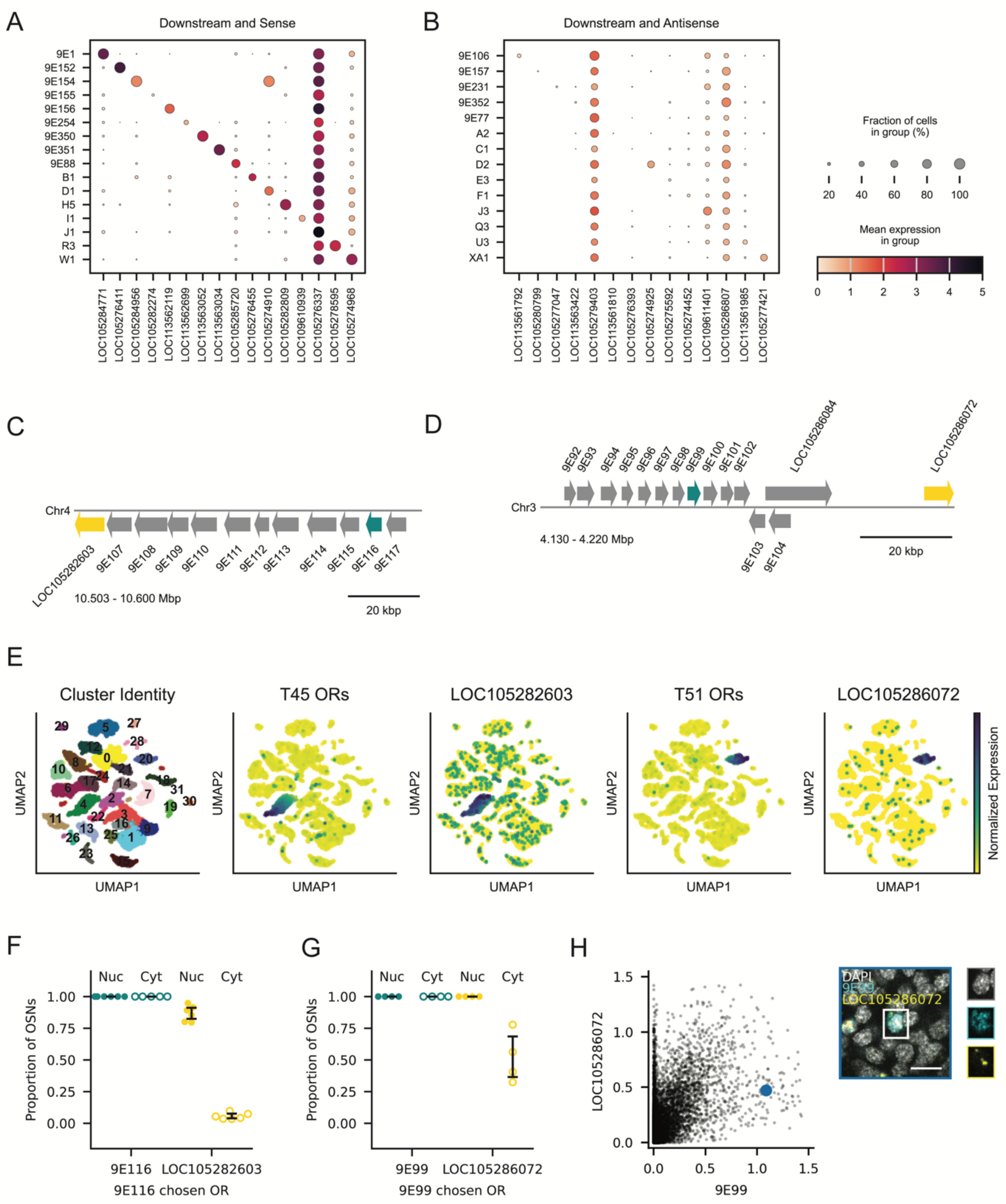

Figure S1. Additional Characterization of Non-OR genes, Related to Figure 1

(A-B) Expression of non-OR genes downstream of singleton ORs on the same strand (A) or opposite strand (B) as the focal OR. Column *n* corresponds to the downstream gene of the singleton OR in row *n*. Each group is composed of cells that express the OR as the chosen OR. Dot size corresponds to percentage of cells in each group that express a gene at a detectable level (>0) and dot color reflects the log-normalized expression level.

bioRxiv preprint doi: https://doi.org/10.1101/2025.08.21.671318; this version posted August 21, 2025. The copyright holder for this preprint (which was not certified by peer review) is the author/funder, who has granted bioRxiv a license to display the preprint in perpetuity. It is made available under aCC-BY 4.0 International license.

(C) Schematic of a subset of T45 highlighting 9E116 (cyan) and LOC105282603 (yellow). 9E116 is located 81 kbp upstream of LOC105282603.

(D) Schematic of a subset of T51 highlighting 9E99 (cyan) and LOC105286072 (yellow). 9E99 is located 51 kbp upstream of LOC105286072.

(E) UMAPs of antennal neurons colored by cluster (left), mean expression of T45 ORs (second from left), expression of LOC105282603 (middle), mean expression of T51 ORs (second from right), expression of LOC105286072 (right).

(F) Proportion of OSNs with 9E116 as the chosen OR per antenna (n=6) exhibiting 9E116 and LOC105282603 signal in the nucleus and cytoplasm.

(G) Proportion of OSNs with 9E99 as the chosen OR per antenna (n=4) exhibiting 9E99 and LOC105286072 signal in the nucleus and cytoplasm.

Error bars: 95% CI centered on the mean (F, G).

(H) Normalized nuclear signal for LOC105286072 vs. 9E99 in segmented OSN nuclei from n=4 antennae. The image with a blue border shows an example cell with nuclear 9E99 and cytoplasmic LOC105286072 that is labeled in blue in the plot. Each channel is shown individually to the right. Scale bar: 5 μm.

bioRxiv preprint doi: https://doi.org/10.1101/2025.08.21.671318; this version posted August 21, 2025. The copyright holder for this preprint (which was not certified by peer review) is the author/funder, who has granted bioRxiv a license to display the preprint in perpetuity. It is made available under aCC-BY 4.0 International license.

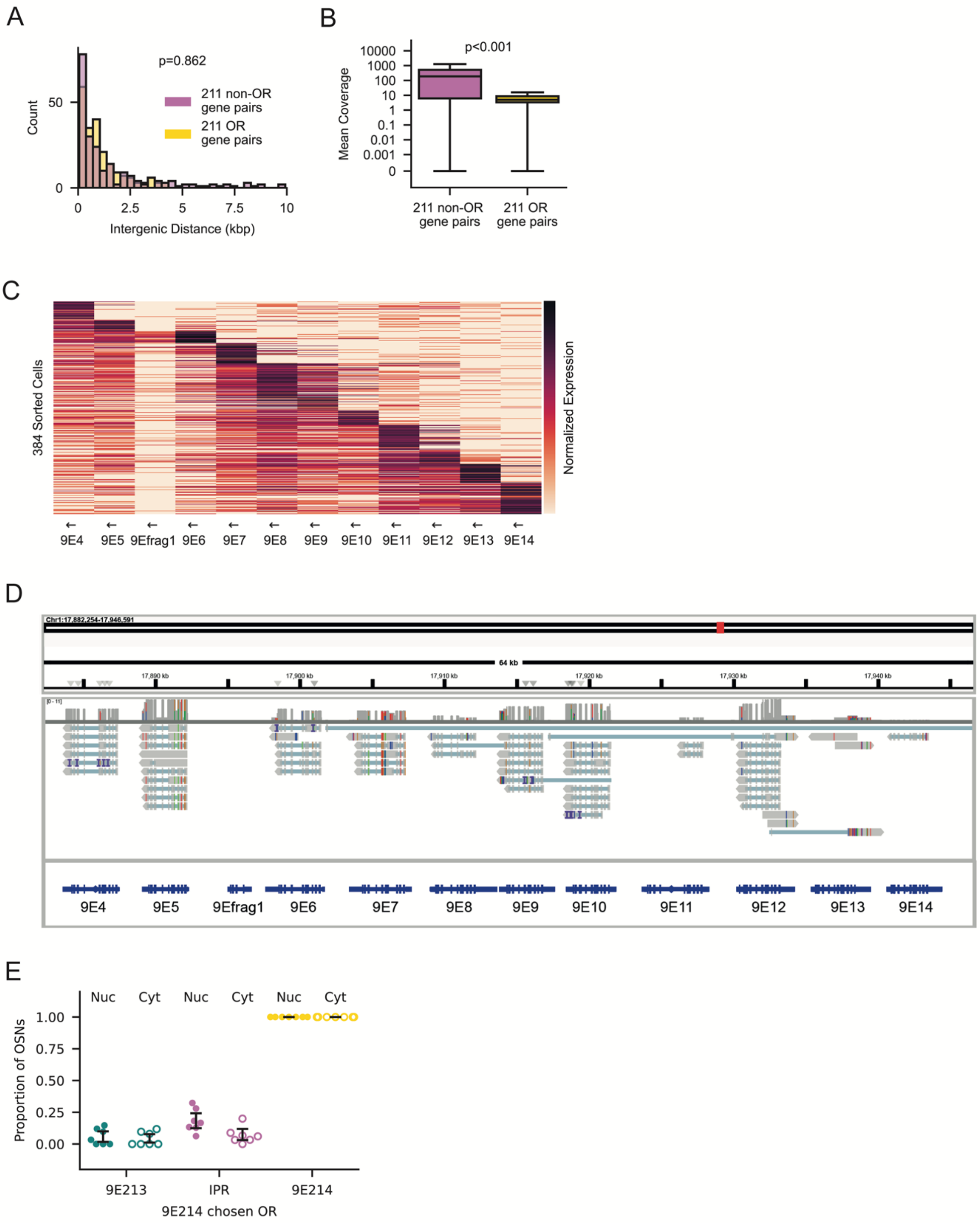

Figure S2. Additional Analysis of Intergenic Regions, Related to Figure 2

(A) Histogram of intergenic distances for 211 non-OR gene pairs (magenta) and 211 OR gene pairs (yellow). P-value from Wilcoxon rank-sum test.

(B) Mean rRNA-depleted RNA-seq coverage across exons of 211 non-OR (magenta) and 211 OR (yellow) gene pairs. P-value from Wilcoxon rank-sum test.

(C) Heatmap of log-normalized expression of all ORs in T79 across cells with a chosen OR in T79. Cells (rows) are sorted by the genomic position of their chosen OR. Arrows indicate strand orientation.

bioRxiv preprint doi: https://doi.org/10.1101/2025.08.21.671318; this version posted August 21, 2025. The copyright holder for this preprint (which was not certified by peer review) is the author/funder, who has granted bioRxiv a license to display the preprint in perpetuity. It is made available under aCC-BY 4.0 International license.

(D) Alignment of long-read mRNA sequencing to the T79 locus.

(E) Proportion of OSNs with 9E214 as the chosen OR per antenna (n=5) exhibiting 9E213, intergenic PR and 9E214 signal in the nucleus and cytoplasm. Error bars: 95% CI centered on the mean.

bioRxiv preprint doi: https://doi.org/10.1101/2025.08.21.671318; this version posted August 21, 2025. The copyright holder for this preprint (which was not certified by peer review) is the author/funder, who has granted bioRxiv a license to display the preprint in perpetuity. It is made available under aCC-BY 4.0 International license.

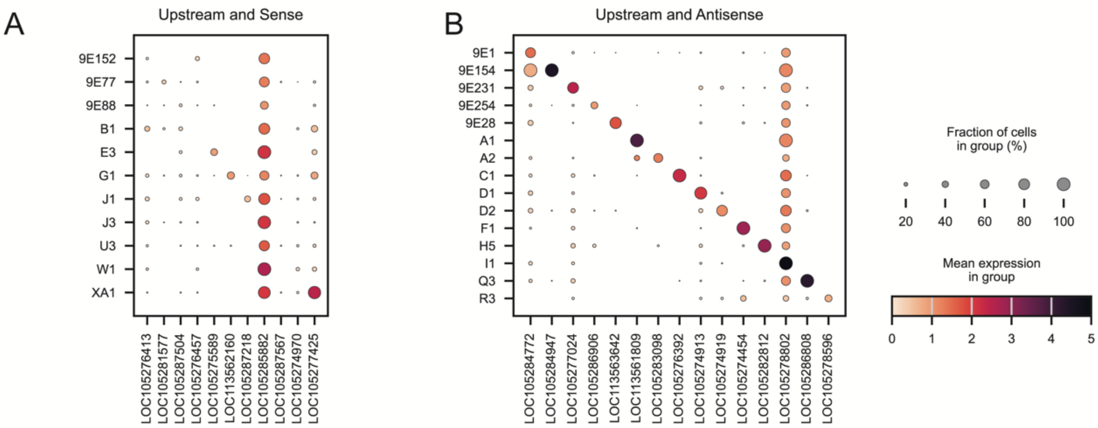

Figure S3. Characterization of Non-ORs Upstream of Singleton ORs, Related to Figure 3

(A-B) Expression of non-OR genes upstream of singleton ORs on the same strand (A) or opposite strand (B) as the focal OR. Column *n* corresponds to the upstream gene of the singleton OR in row *n*. Each group is composed of cells that express the OR as the chosen OR. Dot size corresponds to percentage of cells in each group that express a gene at a detectable level (>0) and dot color reflects the log-normalized expression level.

bioRxiv preprint doi: https://doi.org/10.1101/2025.08.21.671318; this version posted August 21, 2025. The copyright holder for this preprint (which was not certified by peer review) is the author/funder, who has granted bioRxiv a license to display the preprint in perpetuity. It is made available under aCC-BY 4.0 International license.

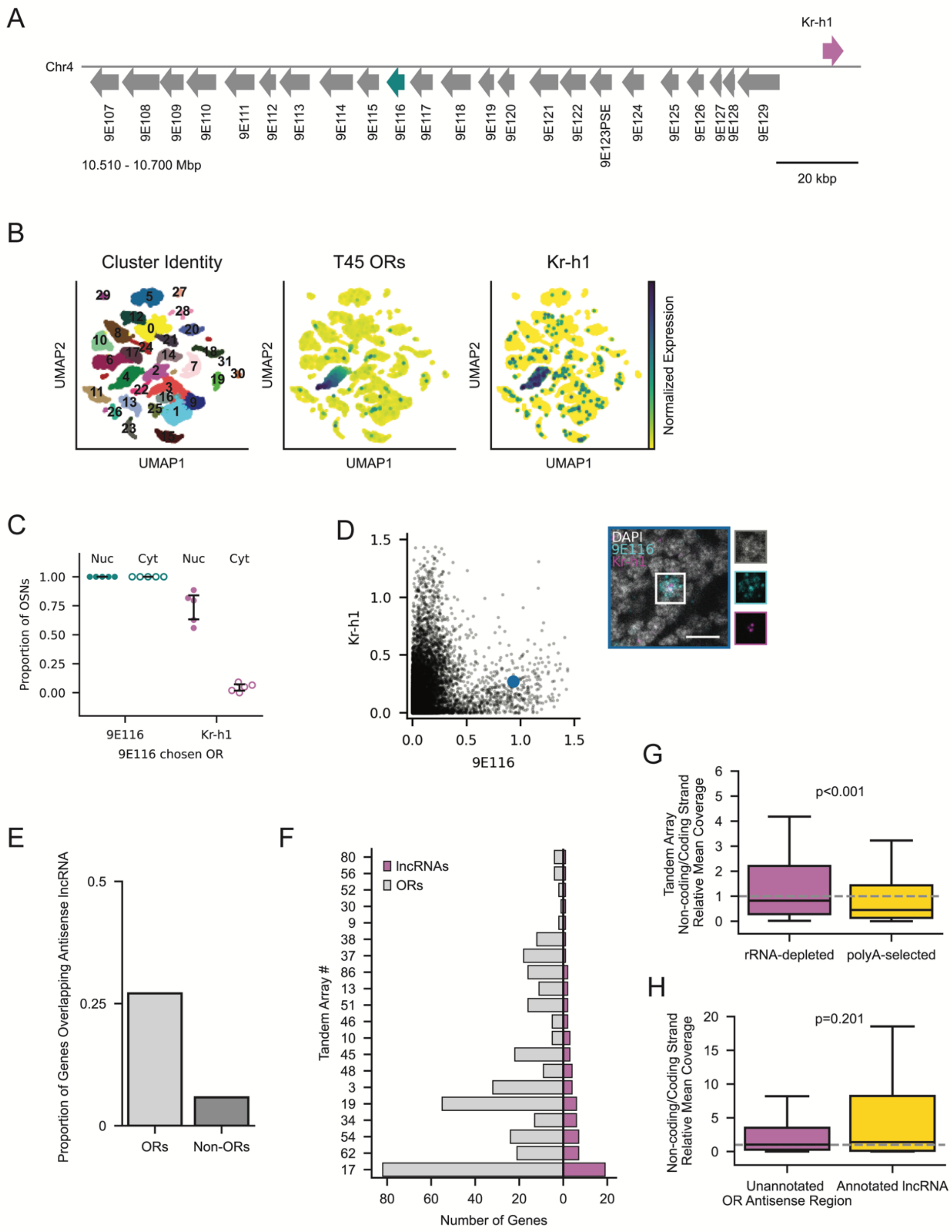

Figure S4. RNA from Upstream Antisense Non-OR Genes is Sequestered, Related to Figure 3

(A) Schematic of T45 highlighting 9E116 (cyan) and Kr-h1 (magenta). 9E116 is located 106 kbp upstream of Kr-h1.

(B) UMAPs of antennal neurons colored by cluster (left), mean expression of T45 ORs (middle), and expression of Kr-h1 (right).

(C) Proportion of OSNs with 9E1116 as the chosen OR per antenna (n=5) exhibiting 9E116 and Kr-h1 signal in the nucleus and cytoplasm. Error bars: 95% CI centered on the mean.

bioRxiv preprint doi: https://doi.org/10.1101/2025.08.21.671318; this version posted August 21, 2025. The copyright holder for this preprint (which was not certified by peer review) is the author/funder, who has granted bioRxiv a license to display the preprint in perpetuity. It is made available under aCC-BY 4.0 International license.

(D) Normalized nuclear signal for Kr-h1 vs. 9E116 in segmented OSN nuclei from n=5 antennae. The image with blue borders shows an example cell with cytoplasmic 9E116 and nuclear Kr-h1 that is labeled in blue in the plot. Each channel is shown individually to the right. Scale bar: 5 µm.

(E) Proportion of OR and non-OR genes that overlap with annotated antisense lncRNAs.

(F) Number of antisense lncRNAs nested within each OR tandem array (magenta), and the number of OR genes per array (grey).

(G) Ratio of non-coding to coding strand coverage for tandem arrays with ≥2 OR genes, using rRNA-depleted RNA-seq (magenta) and polyA-enriched RNA-seq (yellow).

(H) Relative coverage of OR antisense regions (magenta) and annotated antisense lncRNAs nested in tandem arrays (yellow). Antisense coverage is normalized to the coding-strand coverage.

(G, H) Each boxplot shows the median and quartiles; the whiskers extend to 1.5 times the interquartile range. P-value from Wilcoxon rank-sum test. Dotted line at y=1.

bioRxiv preprint doi: https://doi.org/10.1101/2025.08.21.671318; this version posted August 21, 2025. The copyright holder for this preprint (which was not certified by peer review) is the author/funder, who has granted bioRxiv a license to display the preprint in perpetuity. It is made available under aCC-BY 4.0 International license.

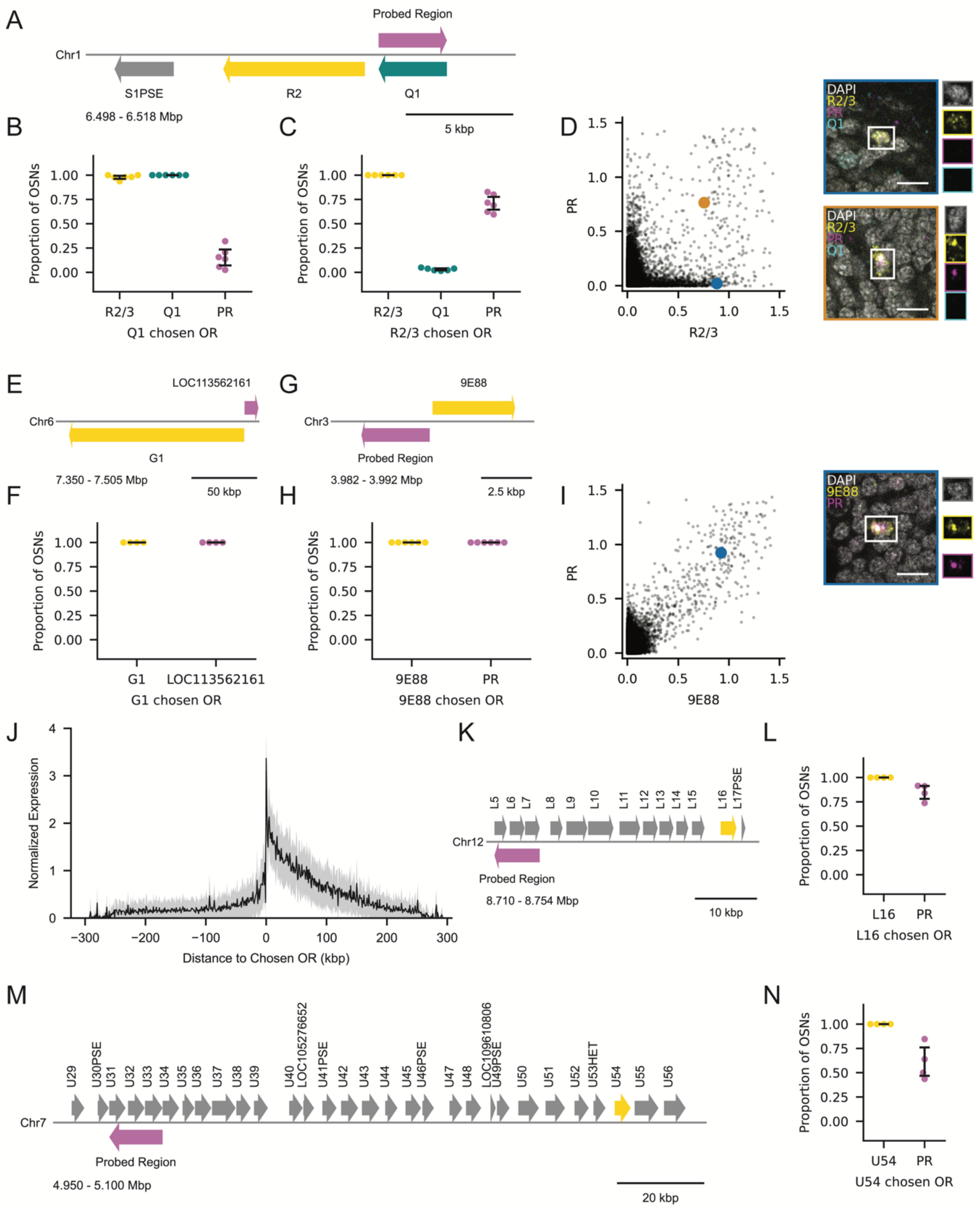

Figure S5. Additional Staining of lncRNAs, Related to Figure 4

(A) Schematic of T70, highlighting Q1 (cyan), R2 (yellow), and the probed region (PR) targeting a putative antisense lncRNA (magenta).

(B-C) Proportion of OSNs with Q1 (B) or R2/3 (C) as the chosen OR per antenna (n=6) exhibiting Q1, R2/3 and PR signal in the nucleus.

(D) Normalized nuclear signal for PR vs. R2/3 in segmented OSN nuclei from n=6 antennae. Images with colored borders reflect the cells labeled with the corresponding colors and each

bioRxiv preprint doi: https://doi.org/10.1101/2025.08.21.671318; this version posted August 21, 2025. The copyright holder for this preprint (which was not certified by peer review) is the author/funder, who has granted bioRxiv a license to display the preprint in perpetuity. It is made available under aCC-BY 4.0 International license.

channel is shown individually to the right of each image. Blue: cell with only cytoplasmic R2/3. Orange: cell with cytoplasmic R2/3 and nuclear PR.

(E) Schematic of the singleton OR G1 (yellow) and the antisense lncRNA LOC113562161 (magenta). G1 is 48 kbp away from the nearest other OR.

(F) Proportion of OSNs with G1 as the chosen OR per antenna (n=4) exhibiting G1 and LOC113562161 signal in the nucleus.

(G) Schematic of 9E88 and a probed region (PR) targeting a putative antisense lncRNA (magenta).

(H) Proportion of OSNs with 9E88 as the chosen OR per antenna (n=6) exhibiting 9E88 and PR signal in the nucleus.

(I) Normalized nuclear signal for 9E88 vs. PR in segmented OSN nuclei from n=6 antennae. The blue dot indicates an example cell with cytoplasmic 9E88 and nuclear PR. The cell is shown in the blue-ordered image, and each channel is shown individually to the right.

(J) Mean and standard deviation of OR expression vs. genomic distance from the chosen OR TSS using snRNA-seq data.

(K) Schematic of a subset of T3, highlighting L16 (yellow) and the probed region (PR) targeting a putative antisense lncRNA (magenta) 30 kbp upstream.

(L) Proportion of OSNs with L16 as the chosen OR per antenna (n=4) exhibiting L16 and PR signal in the nucleus.

(M) Schematic of a subset of T19, highlighting U54 (yellow) and the probed region (PR) targeting a putative antisense lncRNA (magenta) 103 kbp upstream.

(N) Proportion of OSNs with U54 as the chosen OR per antenna (n=4) exhibiting U54 and PR signal in the nucleus.

Error bars: 95% CI centered on the mean. Scale bars: 5 μm.

bioRxiv preprint doi: https://doi.org/10.1101/2025.08.21.671318; this version posted August 21, 2025. The copyright holder for this preprint (which was not certified by peer review) is the author/funder, who has granted bioRxiv a license to display the preprint in perpetuity. It is made available under aCC-BY 4.0 International license.

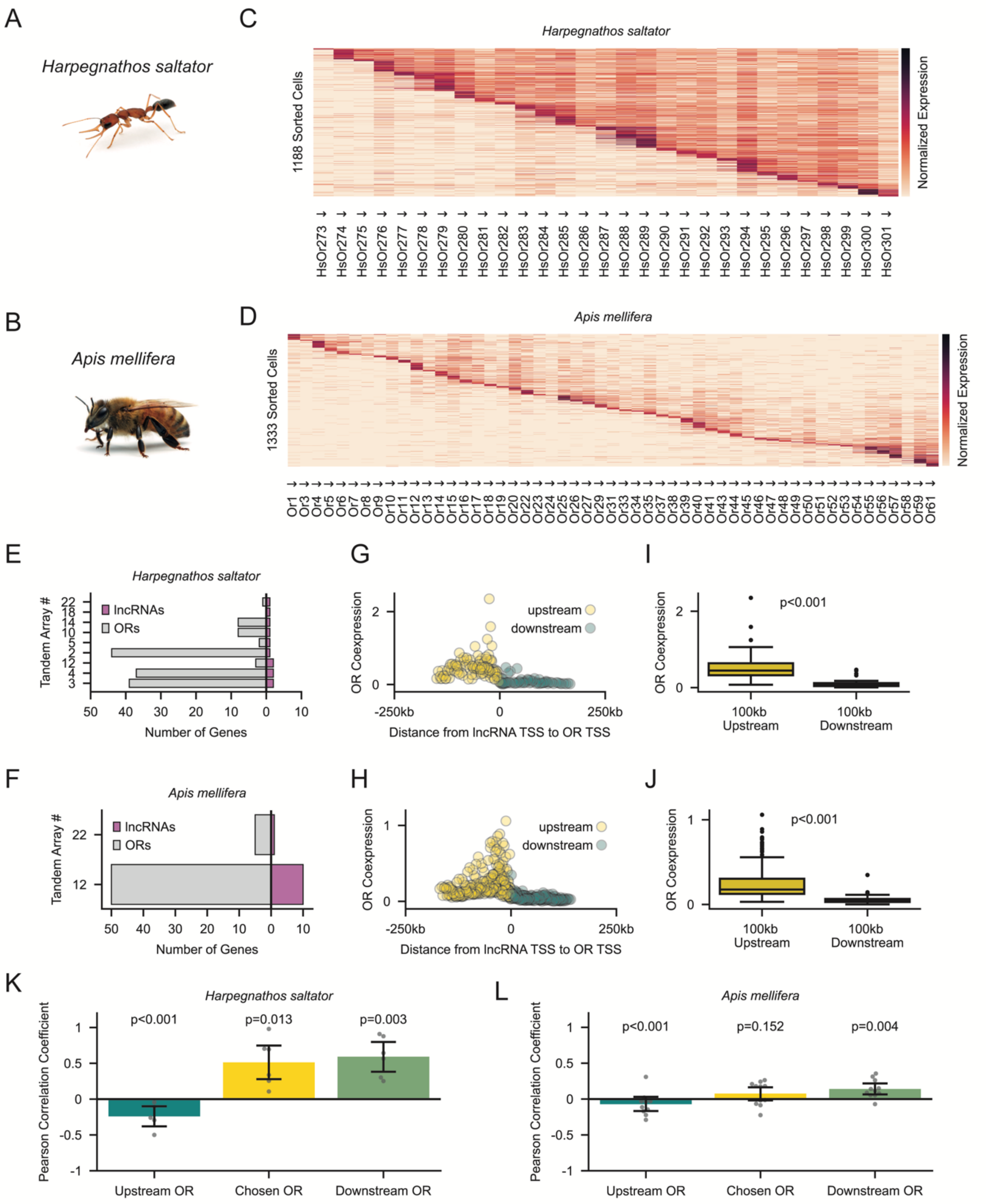

Figure S6. Bidirectional Promoter Activity in Other Ants and Bees, Related to Figure 5

(A-B) Photographs of *Harpegnathos saltator* (A) and *Apis mellifera* (B) workers (images by Alex Wild).

(C-D) Representative tandem arrays from *H. saltator* (C; 30 ORs) and *A. mellifera* (D; 53 ORs). Heatmaps of log-normalized expression of all ORs in each tandem array across cells with a chosen OR in the corresponding tandem array. Cells (rows) are sorted by the genomic position of their chosen OR. Arrows indicate strand orientation.

(E-F) Number of antisense-annotated lncRNAs (magenta) nested within each tandem array and the corresponding number of ORs per array (grey) in *H. saltator* (E) and *A. mellifera* (F).

bioRxiv preprint doi: https://doi.org/10.1101/2025.08.21.671318; this version posted August 21, 2025. The copyright holder for this preprint (which was not certified by peer review) is the author/funder, who has granted bioRxiv a license to display the preprint in perpetuity. It is made available under aCC-BY 4.0 International license.

(G-H) Mean log-normalized coexpression of upstream (yellow) and downstream (cyan) ORs vs. the TSS-TSS distance from lncRNAs, using antennal snRNA-seq data from *H. saltator* (G) and *A. mellifera* (H). Each dot represents a cell in which the corresponding lncRNA is detected.

(I-J) Boxplots of log-normalized OR coexpression within 100 kbp upstream (yellow) or downstream (cyan) of nested antisense lncRNAs, using antennal snRNA-seq data from *H. saltator* (I) and *A. mellifera* (J). Each boxplot shows the median and quartiles; the whiskers extend to 1.5 times the interquartile range. P-values from Wilcoxon rank-sum tests.

(K-L) Pearson correlation coefficients for each unique lncRNA and either upstream ORs (cyan), chosen ORs (yellow), or downstream ORs (green), using antennal snRNA-seq data from *H. saltator* (K) and *A. mellifera* (L). Each lncRNA has a 3’ end within ≤5 kb of the chosen OR TSS. P-values from one-sample t-tests against zero. Error bars: 95% CI centered on the mean.

bioRxiv preprint doi: https://doi.org/10.1101/2025.08.21.671318; this version posted August 21, 2025. The copyright holder for this preprint (which was not certified by peer review) is the author/funder, who has granted bioRxiv a license to display the preprint in perpetuity. It is made available under aCC-BY 4.0 International license.

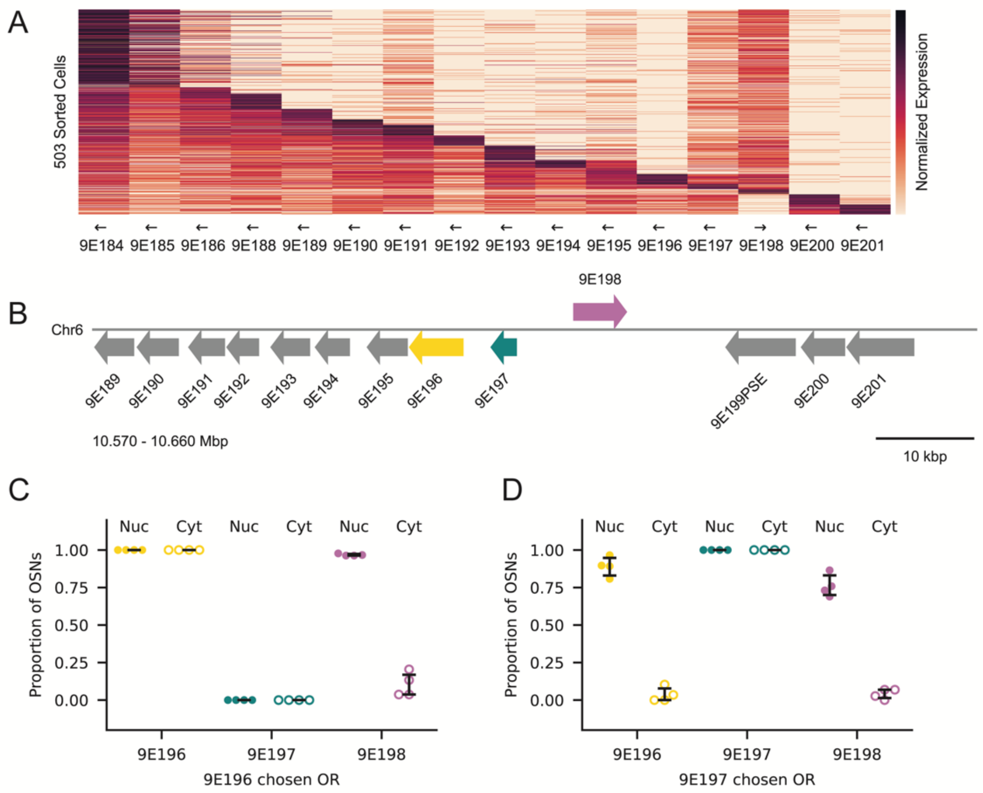

Figure S7. Additional Staining of Inverted OR Genes, Related to Figure 7.

(A) Heatmap of log-normalized expression of all ORs in T35 across cells with a chosen OR in T35. Arrows indicate strand orientation.

(B) Schematic of a subset of T35, highlighting ORs 9E196 (yellow), 9E197 (cyan), and 9E198 (magenta).

(C–D) Proportion of OSNs with 9E196 (C) or 9E197 (D) as the chosen OR per antenna (n=4) exhibiting 9E196, 9E197 and 9E198 signal in the nucleus and cytoplasm. Error bars: 95% CI centered on the mean.
